# An Electrical Method to Detect Both Crack Creation and Propagation in Solid Electrical Insulators †

**DOI:** 10.3390/ma18010024

**Published:** 2024-12-25

**Authors:** Tara Niakan, Zarel Valdez-Nava, David Malec

**Affiliations:** LAPLACE, Université de Toulouse, CNRS, INPT, UPS, 31077 Toulouse, Francevaldez@laplace.univ-tlse.fr (Z.V.-N.)

**Keywords:** crack creation, crack propagation, dielectric material, fracto-emission, crack detection, mechanical breakdown

## Abstract

Fracto-emission is the ejection of electrons and positive ions from matter undergoing a mechanical fracture. The creation and propagation of fractures in insulating material can generate an electrical signal that can be detected using a sufficiently fast signal recorder. The theoretical equations related to crack creation/propagation that induce an externally electric signal are detailed for two conditions: with and without an external applied electric voltage. Results from an experiment with no externally applied voltage are presented for fibreglass-reinforced epoxy laminate samples, in which current signals ranging from 50 mA to 100 mA are measured in a time frame of 200 ns. The signal-to-noise ratio is high enough to consider that the signal that was recorded is not a measurement artifact. This method may help to identify and track a crack propagating inside dielectric materials.

## 1. Introduction

The creation and/or propagation of cracks in brittle materials has been studied throughout the years [1,2,3,4]. Different approaches have been used to detect them, such as using the acoustic emission, which is based on the radiation of acoustic waves due to the separation of two attached surfaces. This separates the results into the creation of a crack, followed by its propagation. Other detection strategies to track cracks propagating in solids rely on optical methods; however, they are limited to the sample’s surface and cannot detect the phenomena within the volume. In electrical insulating materials, with ionic and/or covalent bonds, the region where the crack is to be created is originally electrically neutral. When a crack is created, it implies the rupture of the atomic bonds. This phenomenon will induce, instantaneously, charges appearing on the facing surfaces that have just been created. This instantaneous apparition of charges during and following a fracture in the solid insulator is called fracto-emission.

This matter separation, or crack generation and its propagation, also releases a pressure wave that can be intercepted by acoustic emission detection methods. Fracto-emission phenomena were first mentioned by Dickinson et al. [5], in which they observed both electron emission (EE) and positive ion emission (PIE) during the fracture of graphite and epoxy. Later, they studied the role of charge separation during fractro-emission [6] for alumina-filled epoxy and SiO_2_. This research group also studied the fracto-emission in fibre-reinforced and particulate-filled composites [7,8]. They observed the photon emission (phE) appearing as strain was applied to the samples for glass/epoxy and other polymers [7]. Dickinson and his group continued their studies on fracto-emission, observing EE, phE, and radio frequency electromagnetic wave emission (RE) during the deformation and fracture of an Al/epoxy interface and crystal MgO [9,10], which led other researchers to further study this phenomena in ceramics [11,12] and brittle materials [2].

Klakken, Dickinson, and Jensen [13] developed an electrical experimental method to detect indirectly the charges induced by fracto-emission. To do so, they used different insulating samples placed between two electrodes. A gap was left between the surface of the samples and the top electrode. This allowed for a dielectric breakdown of the gaseous phase. In their setup, a 3-point bending test bench allowed for strain and the creation of a crack in the material. High voltage was applied to produce breakdown of the gas, then the voltage was lowered just enough to avoid breakdown, while still measuring a current. The simultaneous application of a mechanical force allowed the crack to be propagated. This crack propagation, by fracto-emission, placed some charges in the space between the sample surface and the high-voltage electrode, increasing the total measured current.

Fracto-emission, where phenomena occur due to the propagation of a crack, is accompanied by acoustic emissions (AE). Using a transducer, AE and EE were measured and compared [14], confirming that fracto-emission is generally accompanied by acoustic emission during the propagation of a crack. Acoustic emission has been used for failure prediction in ceramics [15], observing crack propagation [16,17,18,19], the study of fracture toughness [20,21], and crack resistance of ceramics [22]. The fracto-emission and acoustic emission have opened the door to using a new electrical method for the detection of both crack creation and propagation in solid electrical insulators.

In recent years, fracto-emission has been observed during aerosol deposition (AD) [23,24,25]. Fracto-emission is also reported in insulators such as SrTiO_3_ [26] and borosilicate glasses [27], ceramics [28], and polymers [29] that are exposed to wear or undergoing tribological interactions or failure [30,31].

All the previous works related to fracto-emission rely on detection methods that follow acoustic emissions or electromagnetic emissions. The basic principle is to analyse either the pressure wave propagation or the consequences of the charge generation or recombination. Table 1 shows an overview of different detection methods for fracto-emission. This local charge unbalance that is created will be only temporary, as the photon emission, ion emission, and recombination will eventually take place. This transitive phenomenon also opens the path to track the displacement of the charges just after they have been created. This displacement of charges, while brief and evanescent, could also be detected by an electrical detection method.

In this work, we wish to introduce a new method to detect the electrical phenomena induced in the immediate time frame of the creation and propagation of a crack. This electrical detection method could be possible since fracto-emission will influence temporally the surface charge density in the solid insulator. Our method could make a more direct link between mechanical phenomena and an induced electric phenomena, possibly allowing us to track a crack propagating in an insulting/dielectric material. While electrical measurements of an external current have already been achieved to detect fracto-emissions in conducting materials such as Si wafers [32], to our knowledge, the method proposed by our group has never been described or applied to insulating materials.

**Figure 1:**
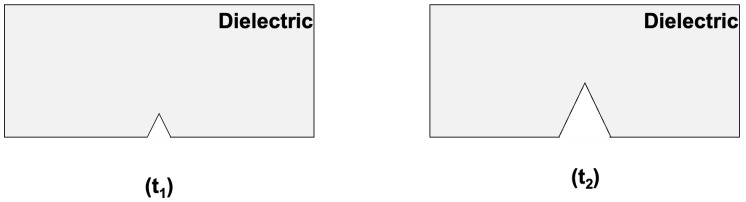
(**Left**) pristine insulator exhibiting a pre-existing crack. (**Right**) propagated crack after the 3PB test.

In the following, we propose to apply a mechanical constraint by using a 3-point bending (3PB) setup in order to create and propagate cracks through a pristine insulator or dielectric material arranged in a parallel capacitor structure. Then, charges that are created and displaced in the volume of the material are sensed by the current induced in the armatures of the capacitor. Figure 1 schematically shows the effects of this constraint as it propagates a crack. Initially, at $t_{1}$, the sample exhibits a pre-existing micro-crack, and using the 3-point bending at $t_{2}$, this crack has propagated, resulting in the insulator matter to separate. A high-frequency amplifier is then connected to track the rapid phenomena while the bending and ultimate breakdown are produced. The theoretical basis of the phenomena is described to give a logical frame for our analysis and method. The results are discussed in terms of repeatability and signal dynamics, trying to give future work perspectives and implications of this work.

**Figure 2:**
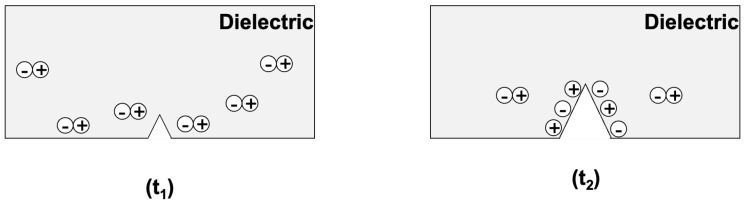
(**Left**) pristine insulator with no charge (neutral). (**Right**) charge separation due to the pre-existing crack propagation.

Simultaneously, during the same event of mater separation, bonds are broken and charge separation is happening (Figure 2).

**Table 1:** Summary of different method of detection of the fracto-emission applied to insulating materials.

Fracto-Emission Detection Method	Acoustic Emission (AE)	Positive Ion Emission (PIE)	Electron Emission (EE)	Photon Emission (phE)	Radio Frequency Electromagnetic Waves Emission (RE)	Electrical Detection
Principle of measurement	Acoustic Transducer or PZT	Channeltron electron multiplier (CEM)	Channeltron electron multiplier (CEM)	Photon counter tube or photomultiplier tube (PMT)	Coil antenna or pickup coils	Electrical acquisition circuit
Reference	[11,13,14]	[5,7,14]	[5,6,7,8,9,10,14]	[6,7,8,9,10]	[6,9,10]	Our team’s work [33]

## 2. Materials and Methods

### 2.1. Insulating Material

**Figure 3:**
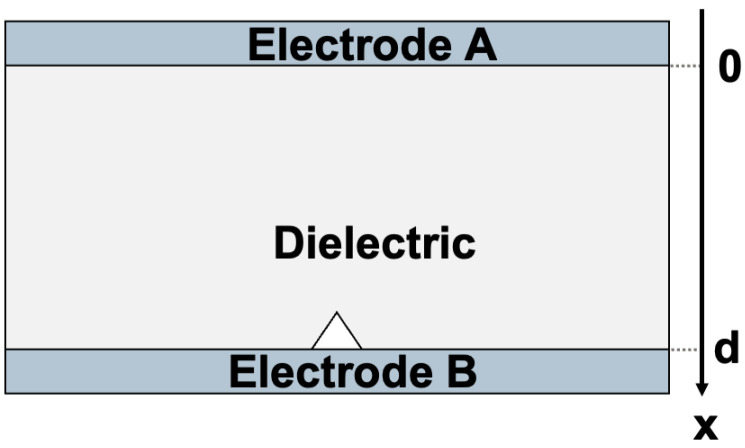
Solid insulator sample with two electrodes.

**Figure 4:**
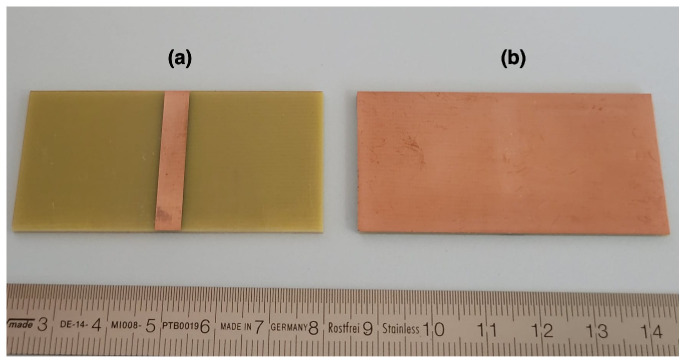
Glass-reinforced epoxy laminate (PCB-FR4), 1 mm thickness, 60 mm length and 30 mm width. The copper metallization on both side has a thickness of 35 μm, with (**a**) the top side of the sample and (**b**) the bottom side.

Knowing that the crack propagation is a very fast phenomenon, the time frame of the event will be very short. In the case of homogeneous materials subjected to a bending test, the crack will propagate very rapidly, in what could be detected as a single event. Trying to track such frugal phenomena could be quite challenging from the acquisition point of view. In the case of a composite material, such as a fibre-reinforced material, the crack will propagate in multiple events because the fibres will slow down the overall propagation of the crack, increasing the chances of observing the event. Glass-reinforced epoxy laminate was therefore used in the following tests. As the detection method is an electrical one, the sample would need to be equipped with conductive electrodes, as shown in Figure 3. Individual layers of pre-impregnated (prepreg) fibreglass epoxy (PCL-FRP-370HR Prepreg, Isola Group, Chandler, AZ, USA), were laminated in a hot press with two layers of copper foils to create a capacitive structure. After thermo-compression, the top layer of copper was etched chemically. This allows for limiting the metallized surface to the narrow region where the mechanical stress will be the maximum in the 3PB test bench. The laminated epoxy substrate, also known as PCB-FR4, had a thickness of 1.6 mm, with 600 mm in length and 30 mm in width. A total of nine samples, named S1 through S9, were fabricated. The structure with the copper metallization of 35 μm of thickness is shown in Figure 4.

**Figure 5:**
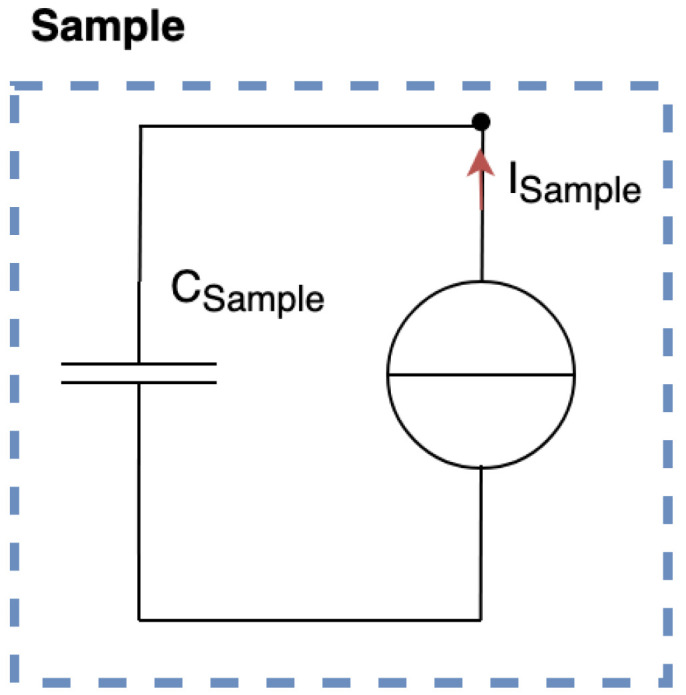
Sample equivalent circuit.

On the following of this study, we want to track the currents generated by a crack being propagated. The sample with the copper armatures could be modelled as a current source and a parasite capacitor named $C_{sample}$, as shown in Figure 5. $C_{sample}$ is the sample capacitance where the dielectric is undamaged, as it not concerned directly with the crack propagation.

### 2.2. Elementary Set-Up for the 3-Point Bending Test

**Figure 6:**
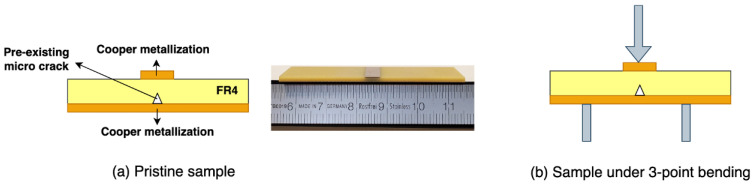
Glass-reinforced epoxy laminate (PCB-FR4) (**a**) Pristine sample with metallization and (**b**) Sample placed under the 3-point bending.

In order to propagate a crack within the material, the sample was placed in a 3-point bending mechanical test bench. The elementary experimental setup is described as follows: (1) the sample is positioned between two electrodes; (2) using a 3-point bending device, an external mechanical constraint is applied to the sample, as shown in Figure 6; (3) applying this external force, a crack is propagated through the sample until the mechanical breakdown is finally achieved. The electrical detection system is described in a later section, since some theoretical considerations are needed first to further clarify how the currents could be generated by this simple mechanical test.

### 2.3. Theoretical Framework for the Current Generation by Electro-Fracture in an Insulator

We propose to use two electrodes on each side of the tested insulator, connected to a fast detection circuit. Considering that the surface charge density evolves as the current passes through the circuit, this current could be measured by a detection circuit that is fast and sensitive enough. The challenge of this approach is to establish the relationships between the surface charge density (a result of Maxwell’s equations), the internal charges generated and displaced during fracto-emission, and also the external applied voltage (V) that could be applied to the sample. First, in order to study the surface charge density $\sigma_{A}$ and $\sigma_{B}$ in the electrodes, two different cases are analysed:

i The sample is short-circuited (V = 0);
ii an external voltage is applied to the sample (V ≠ 0).

In both cases, three fundamental hypotheses were admitted:

1. The sample structure is homogeneous.
2. The sample thickness is sufficiently thin compared to its surface to use a unidimensional formulation.
3. The edge effects are neglected.

The surface charge density of *electrode A* will be called: $\sigma_{A}$ and for *electrode B*: $\sigma_{B}$. The theoretical relationship between surface charge density and the internal charges for a short-circuited charged sample (V = 0) is as follows:

(1) $$\sigma_{A_{\left(E=0\right)}}=\sigma_{B_{\left(E=0\right)}}=\frac{\epsilon_{0}\epsilon_{r}V_{0}}{d}$$

where $\epsilon_{0}$ is the vacuum permittivity, $\epsilon_{r}$ is the relative permittivity, *d* is the sample thickness, and $V_{0}$ is the equivalent voltage needed to be applied to the sample in order to eliminate the charge density at its surface.

When an external voltage is applied (V ≠ 0), the surface charge density becomes:

(2) $$\sigma_{A_{\left(E\neq 0\right)}}=\sigma_{B_{\left(E\neq 0\right)}}=\frac{\epsilon_{0}\epsilon_{r}\left(V_{0}-V\right)}{d}$$

The study of the notion of $V_{0}$, which is the equivalent of the applied voltage to the sample, will be done in the following section.

#### 2.3.1. Relationship Between Surface Charge Density and $V_{0}$

The value of $V_{0}$ is crucial to understanding the parameters that are involved in the magnitude of the surface charge density and its evolution.

The electrical displacement D(x) is given by:

(3) $$D\left(x\right)=\epsilon_{0}\epsilon_{r}E\left(x\right)+P\left(x\right)$$

With E(x) the electrical field and P(x) the polarization. Therefore, the electrical field will be

(4) $$E\left(x\right)=\frac{D(x)-P(x)}{\epsilon_{0}\epsilon_{r}}$$

The integral of the electrical field over the thickness of the sample results in the equivalent applied voltage to the sample:

(5) $$V_{0}=-\int_{0}^{d}E\left(x\right)dx$$

By replacing the electrical field E(x) (4) in (5), we obtain the following:

(6) $$V_{0}=-\frac{1}{\epsilon_{0}\epsilon_{r}}\int_{0}^{d}D\left(x\right)dx+\frac{1}{\epsilon_{0}\epsilon_{r}}\int_{0}^{d}P\left(x\right)dx$$

The charge displacement can be linked to the volumetric charge density ρ(x) by Poisson’s equation [34]: $\rho \left(x\right)=\frac{\partial D(x)}{\partial x}$ in (6); then $V_{0}$ can be

(7) $$V_{0}=-\frac{1}{\epsilon_{0}\epsilon_{r}}\int_{0}^{d}\left(\int_{0}^{x}\rho \left(u\right)du\right)dx+\frac{1}{\epsilon_{0}\epsilon_{r}}\int_{0}^{d}P\left(x\right)dx$$

Using the partial integration method, $V_{0}$ can also be expressed in more detail as

(8) $$V_{0}=-\frac{1}{\epsilon_{0}\epsilon_{r}}\left(d\int_{0}^{d}\rho \left(x\right)dx-\int_{0}^{d}x\rho \left(x\right)dx\right)+\frac{1}{\epsilon_{0}\epsilon_{r}}\int_{0}^{d}P\left(x\right)dx$$

On the other hand, the total charge Q contained in the sample is given by integrating the charge density on the sample thickness:

(9) $$Q=S\int_{0}^{d}\left|\rho \left(x\right)\right|dx$$

with *S* being the surface of the electrodes.

If we consider <x> as the average depth of charge penetration (as introduced in [34]):

(10) $$<x>=\frac{\int_{0}^{d}x\rho \left(x\right)dx}{\int_{0}^{d}\rho \left(x\right)dx}$$

By replacing (9) and (10) into (8), the final expression of $V_{0}$ becomes:

(11) $$V_{0}=-\frac{1}{\epsilon_{0}\epsilon_{r}}\left(d-<x>\right)\frac{Q}{S}+\frac{1}{\epsilon_{0}\epsilon_{r}}\int_{0}^{d}P\left(x\right)dx$$

This would finally give the expression of the charge density $\sigma_{A}$:

(12) $$\sigma_{A}=-\frac{d-<x>}{d}.\frac{Q}{S}+\frac{1}{d}\int_{0}^{d}P\left(x\right)dx-\frac{\epsilon_{0}\epsilon_{r}}{d}V$$

By supposing that in each plan of the Ox abscissa (defined on Figure 3), the permanent polarization is either 0 or uniform. Therefore, no equivalent charges will result. For more simplification, the charge density ρ(x) will be considered as the contribution of the total charge carriers in the volume $\rho_{charge}\left(x\right)$ and the polarization induced charges $\rho_{pol}\left(x\right)$:

(13) $$\rho \left(x\right)=\rho_{charge}\left(x\right)+\rho_{pol}\left(x\right)$$

On the other hand, if we assume that a non-uniform permanent polarization P(x) exists along the Ox axis, this polarization would be equivalent to a charge distribution of $\rho_{pol}\left(x\right)$, with:

(14) $$\rho_{pol}\left(x\right)=\frac{dP(x)}{dx}$$

Considering this new hypothesis, the expressions of Equations (1) and (2) become, respectively:

(15) $$\sigma_{A_{\left(E=0\right)}}=\sigma_{B_{\left(E=0\right)}}=-\frac{d-<x>}{d}\frac{Q}{S}$$

(16) $$\sigma_{A_{\left(E\neq 0\right)}}=\sigma_{B_{\left(E\neq 0\right)}}=\frac{d-<x>}{d}\frac{Q}{S}-\frac{\epsilon_{0}\epsilon_{r}}{d}V$$

This shows that $V_{0}$ is related to the thickness, d, the average depth charge penetration, <x>, the total charge, Q and the surface of the electrode.

#### 2.3.2. Evolution of the Surface Charge Density with the Creation and/or Propagation of a Crack with No Applied Electric Field

The evolution of the surface charge density (15) with the creation and/or the propagation of a crack without any external electric field can be studied with or without mechanical constraint:

- No external mechanical constraint

The sample is free of charge, i.e., there is an infinitesimal quantity of charges in the dielectric that can be neglected. Hence, ρ(x)=0 for x∈[0, d] since Q=0. This means that the surface charge density $\sigma_{A}=\sigma_{B}=0$.

- Under mechanical constraint which creates a crack

In this category, three different scenarios are possible: (1) a case where a pressure wave is created by a crack that is generated or propagated through the thickness of the material, (2) a case where crack creation and/or propagation instantaneously creates electric charges by fracto-emission, and (3) a case where there is charge recombination.

a. Impact of a pressure wave

**Figure 7:**
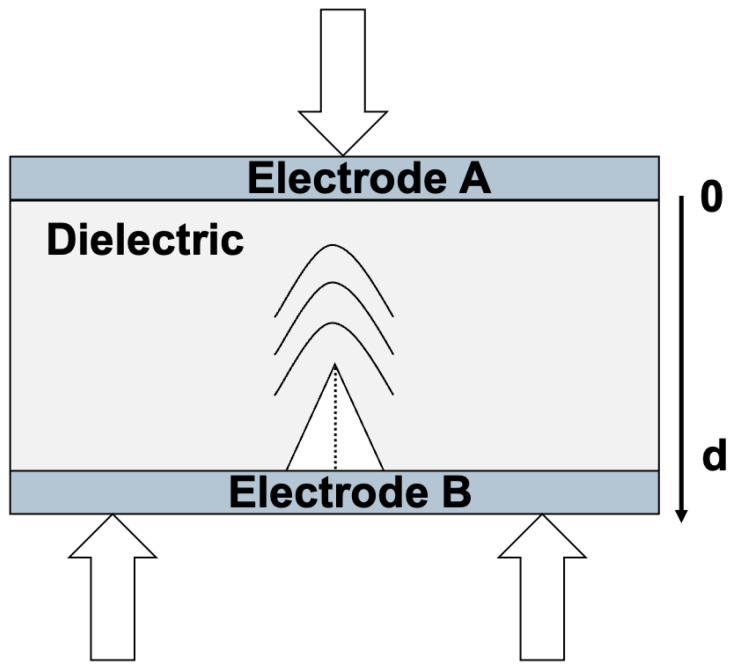
Release of a pressure wave under a mechanical constraint (3-point bending) in the insulator when a crack is created and/or propagates.

Under an external constraint, while a crack is created or propagated, a pressure wave takes place in the insulator (Figure 7).

Equation (9) will be equal to 0 since ρ(x)=0, so Q=0, which results in Equation (15); the surface charge density is also 0. During the release of an induced pressure wave, no electrical signal will be detected.

b. Effect of a crack creating electric charges instantaneously by fracto-emission

**Figure 8:**
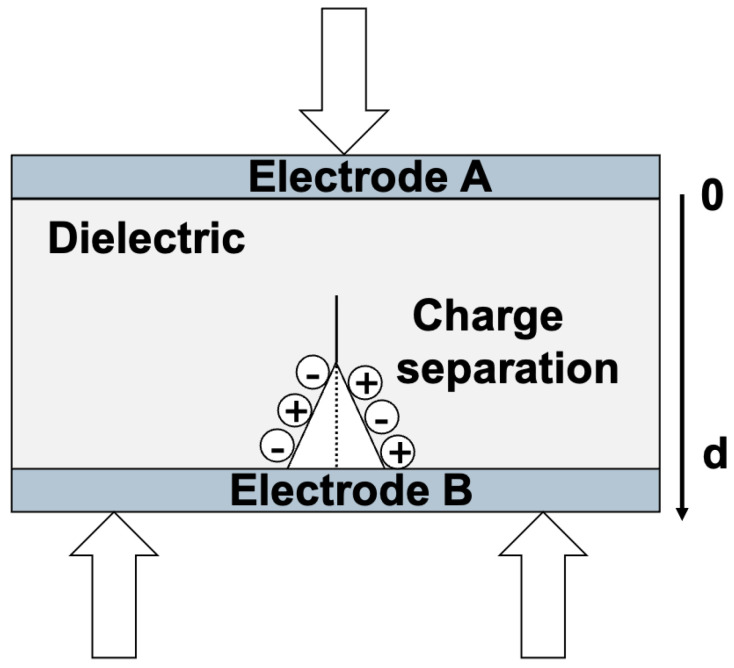
Fracto-emission by the creation of a crack in the dielectric under a 3-point bending test.

The phenomenon of fracto-emission is the instantaneous apparition of charges (Figure 8), so that ρ(x)≠0, <x>≠0, and Q≠0. Therefore, the surface charge density becomes consequently different from zero. Hence, there is a temporal evolution of the surface charge densities due to crack creation and/or propagation, each time it causes fracto-emission. From now on, we will only focus on the volume consisting of the crack in the insulator, the charge, and the electrodes on each side of the insulator.

c. Effect of a crack creating electric charges instantaneously by fracto-emission, followed by charge recombination

**Figure 9:**
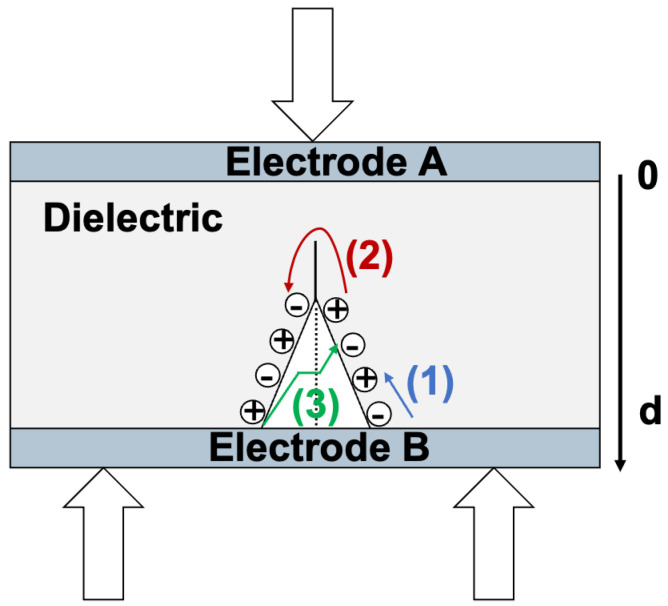
Charges induced by a crack creation and/or propagation. (1) Refers to charge recombination on the crack surface, (2) refers to charge recombination through the volume, and (3) refers to charge recombination across the crack.

During the creation and/or propagation of a crack, the charges created by fracto-emission can initiate a charge recombination, as illustrated in Figure 9. This would mean that the volume charge density will vary. This variation of ρ(x) is followed by the variation of *Q* and <x>. Therefore, $\sigma_{A}$ will also vary.

In Figure 9, (1) refers to charge recombination on the crack surface, while (2) refers to charge recombination through the volume, and (3) refers to charge recombination across the crack. These phenomena will induce a change in surface charge density, leading to an external current. On the other hand, if charge recombination occurs along a plane perpendicular to the d-axis, no external signal will be induced.

We can observe from this theoretical evolution of the surface charge densities that, under mechanical constraints, the creation of a crack creates a fast variation of $\sigma_{A}$ and $\sigma_{B}$.

#### 2.3.3. Surface Charge Density Modification with the Creation and/or Propagation of a Crack with an Applied Electric Field

When an external voltage is applied to the sample, the evolution of the surface charge densities is obtained by using Equation (16). All the previous phenomena of Section 2.3.2 can occur in the dielectric.

**Figure 10:**
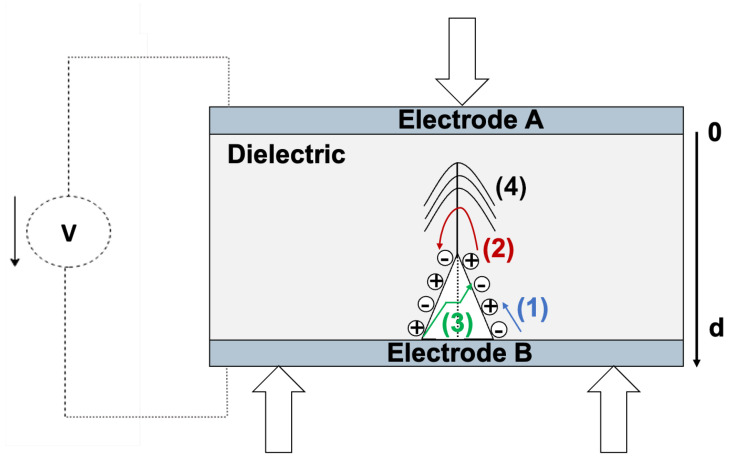
Evolution of the surface charge density with the creation and/or propagation of a crack with an external applied voltage. As seen before, charge recombination can occur (1) on the crack surface, (2) through the volume, or (3) across the crack. A pressure wave (4) can also occur under this condition.

In Figure 10, phenomena (1), (2), and (3) modify *Q* and <x> in the expression of $\sigma_{A}$ (Equation (13)). Whereas phenomenon (4) is the pressure wave created from the crack formation and/or propagation, which modifies $\epsilon_{r}$ and d in $\sigma_{A}$.

Therefore, a crack propagation through an insulator is accompanied by a pressure wave, charge separation, and recombination. These events modify the elements in the expression of the surface charge density, hence modifying $\sigma_{A}$, which could create a fast transient current in the insulator.

### 2.4. Detection Circuit

From the different cases outlined in the previous section, we aimed to detect a crack propagating in the epoxy composite without any applied voltage. The detection circuit must measure fast transient phenomena; hence, a high-frequency acquisition amplifier was employed with a high-pass measuring cutoff. The acquisition circuit has been designed to work without or with the presence of an external electrical voltage [33], as we intend to test the latter configuration in future work.

**Figure 11:**
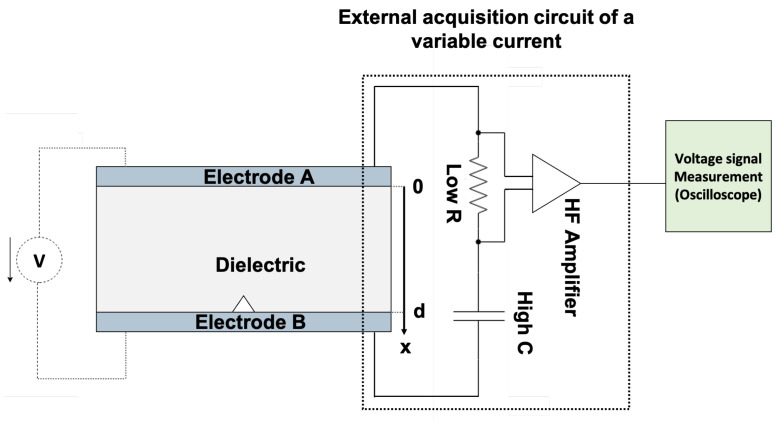
Acquisition circuit.

Figure 11 shows the global structure of the detection circuit. This circuit could detect three phenomena: (1) crack creation and/or propagation, (2) charge recombination, and (3) pressure waves. It is important to note that crack creation and propagation are often accompanied by pressure waves, and in the case where a high voltage is also applied, partial discharges (PD) could also occur.

**Figure 12:**
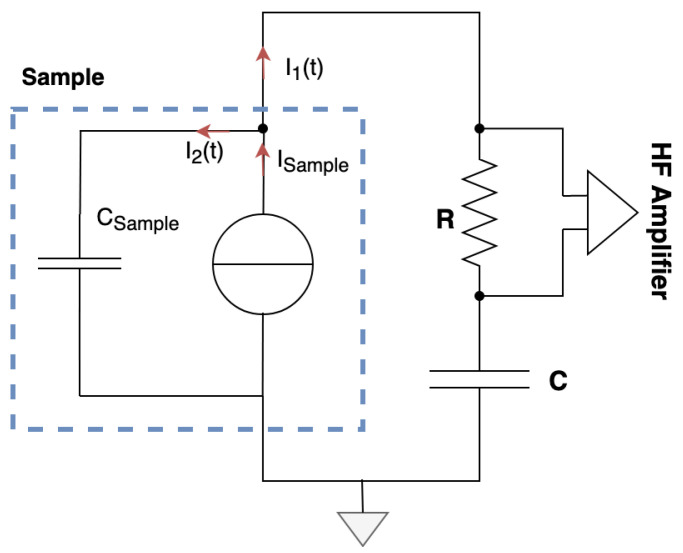
Sample equivalent circuit with the acquisition circuit.

Since we used a current source and a parasite capacitor to model our sample, Figure 12 shows the sample equivalent circuit with the acquisition circuit.

In order for the acquisition circuit to detect the fracto-emission induced current as a result of the creation and/or propagation of a crack, we must make sure that $i_{1}\left(t\right)>i_{2}\left(t\right)$, $i_{1}\left(t\right)$ being the leak current directly linked to the phenomena. This can be obtained if $Z_{sample}>>Z_{C+R}$. Therefore, $C_{sample}$ acts as a parasitic capacitance that absorbs, mainly by dielectric polarization, a part of the crack induced current. The lower the (R+C) high-frequency impedance, the higher the crack induced current ($i_{1}\left(t\right)$) acquired and amplified by the measuring circuit. Hence, as long as the impedance condition, $Z_{sample}>>Z_{C+R}$, is respected, we will be able to observe the leak current.

**Figure 13:**
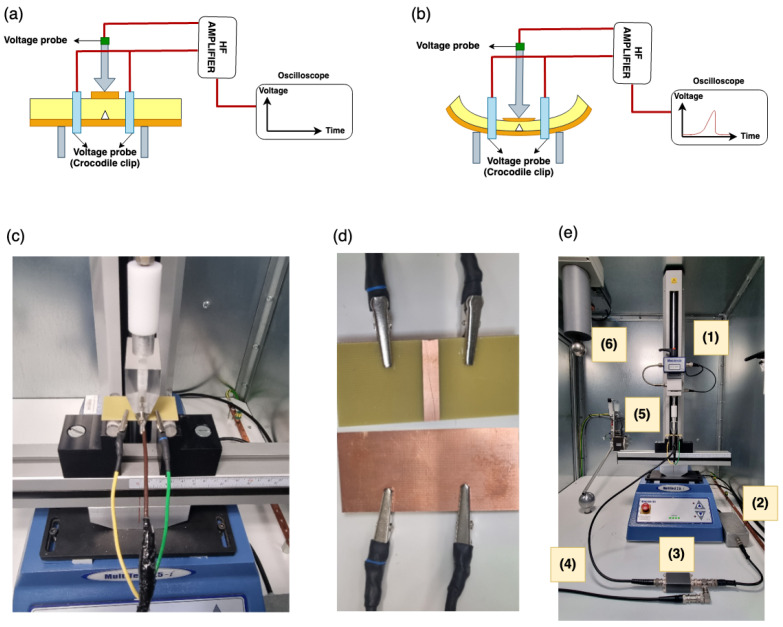
Experimental procedure for the electrical acquisition of the crack propagation: (**a**) Sample positioned under 3-point bending linked to the electric acquisition, (**b**) sample under applied force using the 3-point bending device linked to the electric acquisition, (**c**) sample placed in the 3-point bending device with connecting cable linked to the electric acquisition, (**d**) crocodile clips attached to the sample, upper image, the top side view and bottom image, the back side, (**e**) the experimental setup with (**e1**) the 3-point bending machine, (**e2**) amplifier power supply, (**e3**) HF amplifier, (**e4**) wire between the amplifier output and the oscilloscope, (**e5**) epoxy sample, and (**e6**) AC 50 Hz high voltage for testing under an applied voltage.

All the elements of the experimental setup are shown together in Figure 13. The full setup is composed of the upper loading element of the 3-point bending setup, which is in contact with the upper (smaller) copper metallization. Once the sample is in place, the two cables, yellow and green (left and right cables) in Figure 13c, are attached with crocodile clips to the sample (Figure 13d). The brown cable (central) is plugged into the upper loading element (stainless steel), representing the positive side of the circuit (Figure 13a) and attached to the voltage probe. All three cables are connected to the high-frequency (HF) amplifier, part (a), powered by a constant DC voltage. A “T” connector is used to connect the power supply and the amplifier, and the third one is used to link to the oscilloscope (DPO4054; Tektronix, Beaverton, OR, USA), which is the final output.

After the sample is placed and connected, a force is gradually applied using the 3-point bending machine (Figure 13b). While applying the force, the sample bends, and cracks start to propagate through the epoxy once the critical force is reached. Each of the crack propagations induces an external electric signal that can be seen on the oscilloscope. This signal is the image of the external current circulating in the sample. (Figure 13e) shows an overview of both the experimental setup and the sample under test, with (Figure 13(e1)) showing the 3-point bending machine, (Figure 13(e2)) the amplifier power supply, (Figure 13(e3)) the HF amplifier, (Figure 13(e4)) the wire between the amplifier output and the oscilloscope, (Figure 13(e5)) the epoxy sample, and (Figure 13(e6)) the AC 50 Hz high voltage for testing under an applied voltage.

## 3. Results and Discussion

**Figure 14:**
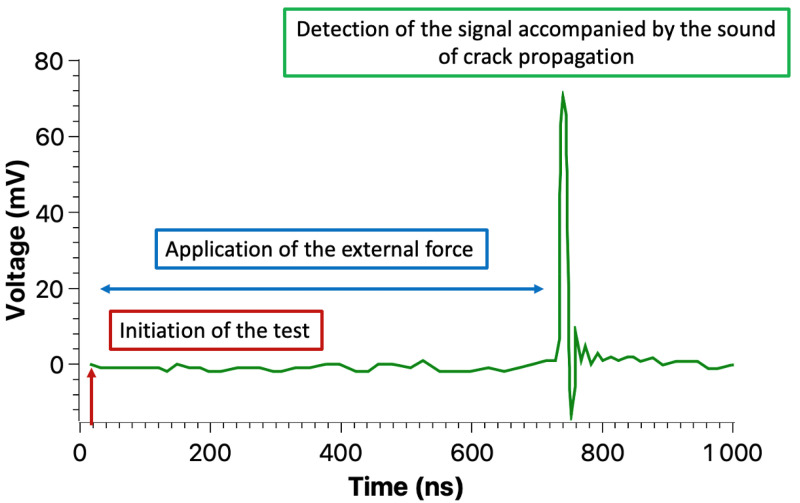
Three phases of the acquisition, (i) the first on being the initialization of the test, (ii) followed by the application of an external force and finally (iii) reaching the critical value to induced a current by propagating a crack (S1).

The tests consisted of applying mechanical force in order to propagate a crack through the insulating material. During the tests, we were able to record the current signal. As shown in Figure 14, initially there is no signal detection, only the noise detected by the acquisition circuit. The signal was only detected once the external force had reached a certain value (between 100 N and 250 N) for which we assume the critical force to propagate a crack has been surpassed.

Each time a signal was detected, the application of the force was stopped, the data of the signal were saved, and then the tests were resumed once again. This procedure continued until the final crack propagation resulted in the mechanical breakdown of the sample. All tests were done using a loading speed of 1 mm/min. The results of 9 samples are shown in the following.

For the samples measured, it was not possible to perform an estimation of the surface charge generated at the armatures of the sample, according to Equation (15), as some parameters cannot be defined experimentally at this stage, such as <x> (the average depth of charge penetration). Nevertheless, because the surface charge is generated in a few ns, a current, our HF amplifier, set to 18.5 dB was sufficient to produce peak signals in the range of 40 mA to 120 mA.

**Figure 15:**
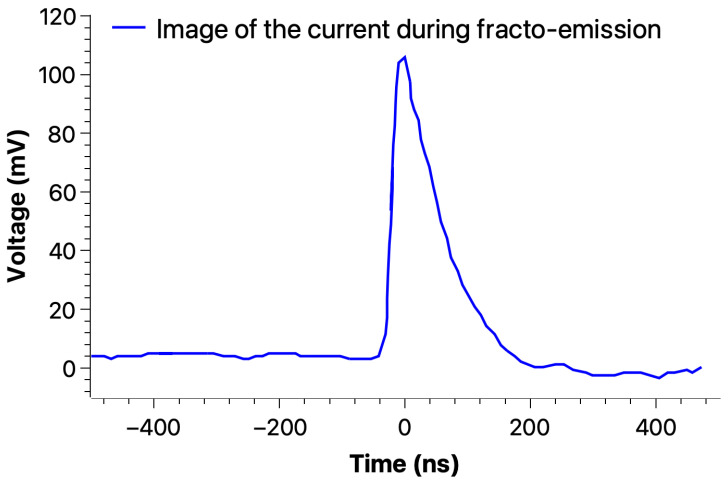
Typical crack-induced current recorded during a 3-point bending (before the final mechanical breakdown) on an epoxy sample (S2).

**Figure 16:**
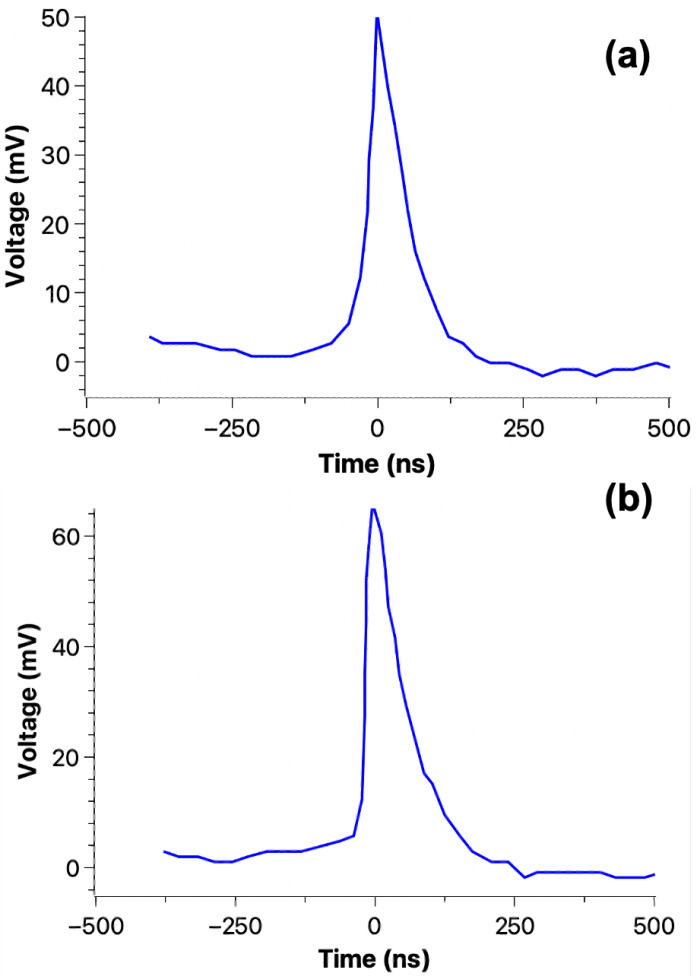
Typical crack-induced currents recorded during a 3-point bending, on an epoxy sample (S3). Both (**a**,**b**) peaks occurred before the final mechanical breakdown.

**Figure 17:**
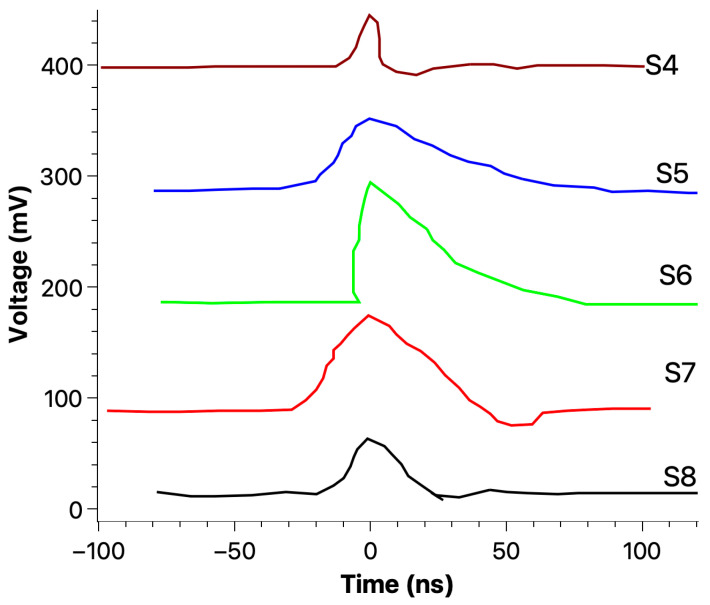
Five crack-induced current signals recorded during the crack propagation using a 3-point bending (before the final mechanical breakdown) on an epoxy sample.

Figure 15, Figure 16 and Figure 17 show the typical fast current recorded during a 3-point bend test on an epoxy sample, indicating that fracto-emission related to (a) crack(s) propagation may be detected by an external electrical circuit.

**Figure 18:**
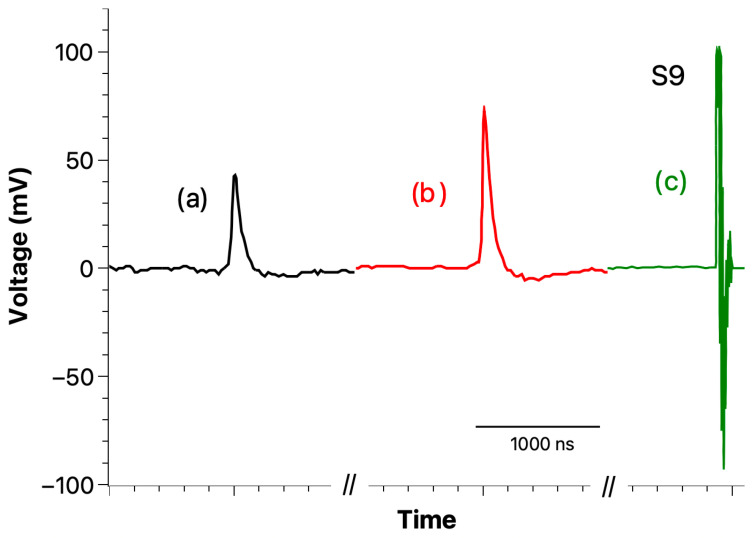
Crack-induced currents recorded during a 3-point bending up to the sample final mechanical breakdown. (a) and (b) are external currents recorded during the test; (c) is the last external current recorded when the sample brakes.

Figure 18 shows three recorded transient currents during the 3-point bending test until reaching the final mechanical breakdown of an epoxy sample. The signal to noise ratio is high enough to consider that the signal that was recorded is indeed a useful signal and not an artifact.

As we can observe in Figure 18, several events can happen during the crack propagation until reaching the mechanical breakdown. It should be mentioned that after each detection the application of force was stopped and then resume once more at the same position.

Our current acquisition circuit has a limitation due to the noise level of the measured signal, which depends on the measurement environment. The size of the sample also plays a detrimental role on the signal to noise ratio; the larger it is, the higher the amount of signal is absorbed by the parasite capacitor $C_{sample}$, therefore part of the information related to the propagation of small and very small cracks is not recorded by the acquisition system.

In future developments, an amplifier with a higher gain could also be used (gain of 32 dB); this could provide a higher intensity of the observed signal, but possibly induce higher levels of noise. The experimental setup could also be automatized, allowing the data acquisition and the application of force to communicate via a programming code. Therefore, once a signal is detected, a flag is generated, stopping the application of the load. Once the data is saved, another command will resume the application of force from the same point it had stopped.

The same acquisition could be possible under high voltage, this time without the external mechanical force, as shown in Figure 11, where the source V will be used to apply the high voltage. This would allow us to observe the effect of the electrical field on the creation and propagation of cracks in dielectric insulators.

## 4. Conclusions and Perspectives

This work proposes the use of an electrical method to identify the creation and propagation of cracks in a solid dielectric. This is accomplished by the analysis of the currents generated by a dielectric sample during a 3-point bending test until breakdown. The currents produced are very small (mA) and produced in short impulses (200 ns). A high-frequency amplifier (800 MHz bandwidth and a gain of 18.5 dB) was able to pick up the signal of a voltage peak systematically at the final mechanical breakdown of the material. This points to charge generation during the fast propagation of a crack within the solid, in accordance with electro-fracture theory. To our knowledge, this is groundwork for the implementation of the fracto-emission phenomena through a pure electrical method. Other pre-breakdown events were also identified and require further study in order to link them to a fast-moving fracture. The proposed measurement method is highly sensitive and requires very little equipment. This method could be a powerful tool for scientific investigation into phenomena involving dielectrics and structural damage from mechanical or combined constraints, e.g., electrical-mechanical, thermal-mechanical, irradiation-mechanical, or chemical-mechanical. On an industrial level, this method can be used to benchmark and test different materials that undergo mechanical stress during operations, so as to identify those likely to cause cracks that reduce service lifetime.

## Data Availability

The original contributions presented in this study are included in the article. Further inquiries can be directed to the corresponding author.
